# Gut microbiome composition is associated with spatial structuring and social interactions in semi-feral Welsh Mountain ponies

**DOI:** 10.1186/s40168-018-0593-2

**Published:** 2018-11-22

**Authors:** Rachael E. Antwis, Jessica M. D. Lea, Bryony Unwin, Susanne Shultz

**Affiliations:** 1 School of Environment and Life Sciences, University of Salford, Salford, UK; 2 School of Earth and Environmental Sciences, University of Manchester, Manchester, UK

**Keywords:** 16S rRNA gene, Amplicon sequencing, Harem, Horizontal transmission, Life stage, Multi-level trait, Spatial proximity, Social networks, Vertical transmission

## Abstract

**Background:**

Microbiome composition is linked to host functional traits including metabolism and immune function. Drivers of microbiome composition are increasingly well-characterised; however, evidence of group-level microbiome convergence is limited and may represent a multi-level trait (i.e. across individuals and groups), whereby heritable phenotypes are influenced by social interactions. Here, we investigate the influence of spatial structuring and social interactions on the gut microbiome composition of Welsh mountain ponies.

**Results:**

We show that semi-feral ponies exhibit variation in microbiome composition according to band (group) membership, in addition to considerable within-individual variation. Spatial structuring was also identified within bands, suggesting that despite communal living, social behaviours still influence microbiome composition. Indeed, we show that specific interactions (i.e. mother-offspring and stallion-mare) lead to more similar microbiomes, further supporting the notion that individuals influence the microbiome composition of one another and ultimately the group. Foals exhibited different microbiome composition to sub-adults and adults, most likely related to differences in diet.

**Conclusions:**

We provide novel evidence that microbiome composition is structured at multiple levels within populations of social mammals and thus may form a unit on which selection can act. High levels of within-individual variation in microbiome composition, combined with the potential for social interactions to influence microbiome composition, suggest the direction of microbiome selection may be influenced by the individual members present in the group. Although the functional implications of this require further research, these results lend support to the idea that multi-level selection can act on microbiomes.

**Electronic supplementary material:**

The online version of this article (10.1186/s40168-018-0593-2) contains supplementary material, which is available to authorized users.

## Background

All metazoan species harbour complex communities of microorganisms referred to as host microbiomes. The host plus its microbiome can be considered as a distinct biological entity, the holobiont, with a complementary genome, the hologenome [1]. Although the concept of a holobiont remains a topic of debate [2, 3], there are several compelling arguments for why this approach is biologically relevant. First, microbiome composition can be heritable such that offspring microbiomes resemble those of their parents [4]. Second, microbiome genomes are much more plastic and evolvable over short periods of time than host genomes, providing hosts with phenotypic plasticity that can respond more rapidly to external and internal challenges [5]. Third, these diverse communities are associated with host functional traits, such as immune function and metabolism, as demonstrated across a range of host sites for both mammalian and non-mammalian taxa [6–8]. For example, microbiome composition and complexity has been associated with disease prevalence for many host taxa at the individual and population level [9–12]. Many host species have a considerable 'core' microbiome that is stable with a body site across individuals, time, and space [13–17]. This core microbiome is thought to represent the heritable "house-keeping" component of the microbiome, while there is also a flexible component to the microbiome that varies based on environmental influences [5]. At the same time, there is significant temporal variation in microbiome composition between and within individuals of a given species [18, 19].

Intriguingly, group-level microbiome similarity may represent an example of a multi-level trait, where heritable phenotypes are influenced by association patterns [20]. In fact, social structure can lead to adaptive and evolutionary changes within the microbiome and, potentially, the host organism [3]. For example, social pollinators, such as honeybees and bumblebees, share a distinct community of bacteria not identified in solitary bee species [21]. The presence of this distinct microbiome provides social bee species with protection from parasitic infection and thus confers fitness benefits not present in solitary species lacking this shared microbiome [22]. In communities, interaction patterns defined by social networks can be used to characterise the nature of interactions between individuals. These networks have long been suggested to impact on transmission dynamics of disease in humans [23, 24] and animals [25, 26], but social interactions should also be associated with the opportunity to share microbiomes and thus may confer fitness benefits [27].

Spatial proximity between individuals has been shown to facilitate microbiome exchange even when social behaviours are minimal. For example, gut microbiomes of solitary North American red squirrels (*Tamiasciurus hudsonicus*; [28]) and mouthpart microbiomes of Phofung river frog tadpoles (*Amietia hymenopus*; [29]) are spatially structured. Similarly, gopher tortoise (*Gopherus polyphemus*) gut microbiome composition is determined by the geographic proximity of burrows, as well as home ranges and kinship [30]. If spatial proximity promotes microbiome similarity, then social structuring determined by patterns of interaction, association and spatial proximity between individuals provides an ideal mechanism for driving subpopulation level patterns in host microbiome communities [31]. The role of social interactions in transmitting pathogens and parasites between individuals is well known; however, such behaviours can also alter and influence the composition of the microbiome [27]. For example, yellow baboon (*Papio cynocephalus*) group membership, social networks, and grooming interactions predict the taxonomic structure of the gut microbiome even after controlling for the effects of diet, kinship and shared environments [32, 33]. Similarly, gut microbiomes of chimpanzees (*Pan troglodytes*) are associated with interaction frequency [34] and human milk microbiomes are influenced by the size of the social network and physical/proximal contact with an infant [35]. Despite the growing interest in the role of social interactions in determining gut microbiome composition [34, 36], the majority of studies focus on primates.

Equids provide an interesting test case for microbiome dynamics at the subpopulation level. As hindgut fermenters, the Equidae are particularly reliant on microbial digestion for energy and nutrition [5, 37]. Free-ranging horses (*Equus ferus caballus*) form harem bands (i.e. family groups) composed of (usually) one mature stallion, multiple mares, and their immature offspring. Found in Snowdonia National Park, the semi-feral Carneddau pony is the closest to a wild unmanaged pony population in the UK [38, 39]. They are direct descendants of the wild Welsh mountain pony and are a genetically unique and distinct population, rendering them a high conservation priority [38]. Over 300 individuals exist within smaller scattered bands that form complex social networks [38–40]. Individuals within bands engage in varying levels of affiliative behaviour with conspecifics dependant on various factors such as kinship, age, social status, and season. Males are socially central (i.e. well connected), while females are more peripheral and tend to have weak bonds with other mares [40]. Although female relationship strength varies between seasons, their position within the social network is stable across years [40].

Here, we determine how the gut microbiome of semi-feral ponies from Snowdonia National Park is influenced by spatial structuring, social interactions, and kin relationships. Using social network analysis combined with 16S rRNA gene amplicon sequencing of faecal samples, we test the following hypotheses: (i) there will be within-individual variation in microbiome composition, but this will not be as large as between-individual variation; (ii) mares will have more similar microbiomes to band stallions than to other mares in their band; (iii) mares will have more similar microbiomes to their own offspring than to other juveniles in the band; (iv) band, life stage, and sex will influence microbiome composition; (v) band-level variation in microbiome composition will be driven by spatial structuring (i.e. social networks).

## Methods

### Study animals

Carneddau Welsh mountain ponies are located in the Carneddau mountain range, Snowdonia National Park, North Wales (53.22°N, 3.95°W) over an area of approximately 35–40 km^2^ of commons land between 287 and 610 m above sea level. The land is used primarily for sheep farming and recreational hiking and thus ponies are habituated to human presence but not to physical contact. The population is essentially unmanaged aside from an annual roundup event each November, during which individuals are herded onto adjacent farmland for 1 to 2 days for population monitoring and management purposes. Individuals can be identified using their age-sex classification and a photographic database that depicts coat colour, face and leg markings, and ear tags/notches. For this study, we collected data from 30 individuals across three focal bands (Aber, Marsh, and Valley) that have been the subjects of long-term behavioural and demographic data collection [39, 40] (Table 1).

**Table 1:** Demographic data for each band used in this study

Band	Adults		Sub-adults		Foals		Total individuals
	Stallion	Mares	Female	Male	Female	Male	
Aber	1	6	0	1	1	0	9
Marsh	1	7	1	0	2	2	13
Valley	1	4	1	1	0	1	8

### Distribution mapping and social network analysis

Demography and proximity data were collected over ten sampling days between the 21 August and 14 November 2014 (the same time period when faecal sampling also occurred). All ponies included in the spatial analyses were sighted a minimum of 5 days, sampled opportunistically within the study area. Upon encountering a group, we recorded time, pony IDs, and GPS location along with an approximate spatial network of the ponies. We plotted the geographic distribution of the bands over the study period using the ggmap package [41] in RStudio (v1.0.153) [42] for R (v3.4.1) [43].

For the social network analysis, we approximated the distance in metres between individuals. All individuals less than ~ 100 m apart and moving as a cohesive unit were considered to be associated with each other [40]. Association matrices were constructed for each day of sampling; individuals that were close together (< 15 m) or interacted were given a score of 2, other individuals (i.e. those 15–100 m apart) were given a score of 1 and more than 100 m apart scored 0. Using these association scores, an overall weighted association index for each dyad was calculated using a modified version of the simple ratio index [44], where edge weight was calculated as:

$$ {E}_{AB}=\frac{x_{\mathrm{SUM}}}{2{x}_{\mathrm{COUNT}}+{y}_{AB}+{y}_A+{y}_B} $$

where *x*_SUM_ is the sum of associations between individuals *A* and *B*, *x*_COUNT_ is the number of times *A* and *B* have been sighted together (where *x*_COUNT_ multiplied by two is the maximum possible association score), *y*_*AB*_ is the number of times both *A* and *B* were observed but not together, *y*_*A*_ is the number of times only individual *A* was seen, and *y*_*B*_ is the number of times only *B* was seen.

### Sample collection and 16S rRNA gene amplicon sequencing

For each band, faecal samples were collected from the stallion plus 4–7 mares and 2–5 juveniles (Table 1 and Additional file 1: Table S1) between the 21st August and 11th November 2014, prior to the annual round-up. Faecal samples were collected using sterile gloves. Most samples were collected within 10 minutes of defecation, but on rare occasions, this took up to a maximum of 1 hour when multiple individuals defecated within a short period. Several samples were collected from different parts of the dung pile, but no faeces in contact with the ground were collected (thus, there was minimal risk of environmental contamination). The samples were mixed thoroughly by hand in a sterile bag and a subsample retained for analysis. Three to five samples were collected per individual across the four study months (Additional file 1: Table S1). Samples were stored and transported in cool bags to the University of Manchester the same day and frozen at − 80 °C prior to DNA extraction.

DNA was extracted using the QIAamp DNA Stool Mini Kit (Qiagen, UK) following the manufacturer’s protocol with an additional incubation time of 30 min at 95 °C. A blank extraction was also included to act as a negative control for sequencing. DNA was amplified for the 16S rRNA gene (v4 region) using dual-indexed forward and reverse primers according to Kozich et al. [45] and Griffiths et al. [29]. Briefly, PCRs were run in duplicate using Solis BioDyne 5x HOT FIREPol® Blend Master Mix, 2 μM primers and 1 μl of sample DNA. Thermocycling conditions were as follows: 95 °C for 15 min; 28 cycles of (95 °C for 20 s, 50 °C for 60 s, 72 °C for 60 s), and a final extension at 72 °C for 10 min. PCR replicates were checked on an Agilent 2200 TapeStation, combined into a single PCR plate and cleaned using HighPrep™ PCR clean up beads (MagBio, USA) according to the manufacturers’ instructions. Products were quality checked using an Agilent 2200 TapeStation and quantified using a Qubit™ 3.0 Fluorometer according to the manufacturers’ protocol. Samples were pooled according to concentrations in order to minimise sequencing bias. Paired-end (2 × 250 bp) amplicon sequencing was conducted on an Illumina MiSeq platform with negative and positive (mock community) controls.

### Pre-processing of microbiome data

We conducted all analyses in RStudio (v1.0.153) [42] for R (v3.4.1) [43]. A total of 3,208,334 raw sequence reads from 112 samples were generated during sequencing. We conducted sequence processing in dada2 v1.5.0 [46] using the default pipeline (see Additional files 1, 2, 3, and 4). Modal contig length was 253 bp once paired-end reads were merged. We removed sequence variants (SVs) with length > 260 bp (4 SVs; 0.086% of total sequences) along with chimeras and two SVs found in the negative controls, leaving an average of 22,294 reads per sample (range 8071–42,869). We assigned taxonomy using the SILVA v128 database [47, 48]. To provide greater taxonomic detail about unidentified SVs and to stop the removal of these during analyses that agglomerate to a given taxonomic level, we fully annotated the taxonomy table to species level using higher levels assignments (e.g. SV1 was named “Family_Prevotellaceae” at the genus and species levels). We exported the final SV table, taxonomy table, and sample metadata to the phyloseq package [49] and converted the data to relative abundance for further analyses.

### Microbiome variation according to ID

We produced an NMDS plot in phyloseq using the Bray-Curtis distance matrix to visualise the variation within and between individuals according to community composition. To determine the microbiome variation attributable to individual variation (ID), we conducted a permutational ANOVA (PERMANOVA; adonis) in the vegan package [50].

We calculated the core microbiome of individual samples using a detection threshold of 0.001% and a prevalence threshold of 99.9% (i.e. a given SV must be present in 99.9% of individuals with a relative abundance of at least 0.001%) in the microbiome package [51]. We used an NMDS plot to visualise the variation in core microbiome according to ID and analysed the data using an adonis analysis (as above).

To determine whether there was greater microbiome variation *within* an individual than *between* individuals, we calculated Jensen-Shannon Divergence (JSD) values in the phyloseq package [49]. JSD values give a measure of similarity between all individual samples (i.e. by calculating the distance between samples) either from the same individual (i.e. within-individual variation) or from different individuals in the same band (i.e. between-individual variation). Smaller JSD values indicate more similar microbial communities and conversely, larger values indicate a less similar community. We used a generalised linear mixed model with ID and band as random factors to compare JSD distances within individuals to JSD distances between individuals and visualised the data using a bar chart.

### Microbiome variation according to band, life stage, and sex

We categorised individuals under the age of 1 year as foals and for older individuals, females < 2 years old and males < 3 years old as sub-adults (females are usually reproductively mature from 2 years onwards but males take longer to mature, disperse, and attract mares). We classified all others as adults, with the exception of one female who still displayed sub-adult behaviours, did not disperse from her natal band, and had not foaled by age 3, and so was considered a sub-adult. We visualised the taxonomic composition (at the class level) of the communities according to band and life stage using stacked plots in phyloseq [49] and ggplot2 [52].

To obtain the “average microbiome” for an individual, we merged raw sample data within an individual using the merge_samples function in phyloseq (using “fun = mean”) [49]. To determine whether there was greater microbiome variation between bands than within bands, we calculated Jensen-Shannon Divergence (JSD) values between individuals using data from their average microbiome, as described above. We used a one-way ANOVA with Tukey’s post hoc analysis to compare JSD distances within and between bands and visualised the data using a bar chart.

We produced NMDS plots in phyloseq using the Bray-Curtis distance matrix to visualise differences in beta diversity according to band and life stage. We used an adonis analysis to test for significant effects of band, life stage, and sex on total microbiome community composition. We then calculated the core microbiome as described above and repeated the adonis analysis for this core community. Additionally, we agglomerated the core taxa to genus level and visualised the core microbiome as a heat map to give a representation of the bacterial taxa present.

To identify differences in microbiome composition between foals (which at approximately 5–8 months old, were most likely still nursing) and sub-adults (which were most likely fully weaned), we conducted an indicator analysis using the multipatt function in the indicspecies package [53].

### Effects of spatial structuring and social interactions on the microbiome

We correlated the social network association matrix with the NMDS scores of each individuals’ average microbiome using Kendall’s correlation coefficient for non-parametric data with ties. Networks were constructed and visualised using the igraph package [54] with edges weighted by either microbiome similarity (the inverse of the NMDS distance) or the association index as described above. As the microbiome distance matrix is fully connected, we delated edges with a similarity less than the mean value for the population. We calculated JSD values between merged samples in the phyloseq package [49] and used general linear mixed models (with ID and band as random factors) to identify whether mares had more similar microbiomes to other mares within the same band or to the band stallion and whether mares had more similar microbiomes to their own offspring than to other mares’ offspring within the same band. We visualised these using bar charts.

## Results

Bacteria primarily belonged to the Bacteroidia, Clostridia, Spirochaetes, and Fibrobacteria classes (Bacteroidetes, Fibrobacteres, Firmicutes, and Spirochaetae phyla) (Additional file 1: Figure S1 and S2). The dominant families represented in the core microbiome were anaerobic bacteria associated with grass-eating mammals, including Prevotellaceae, Ruminococcaceae, Rikenellaceae, Lachnospiraceae, Spirochaetaceae, Fibrobacteraceae, Christensenallaceae, Erysipelotrichaceae, Acidaminococcaceae, and various groups of Bacteroidales.

**Fig. 1:**
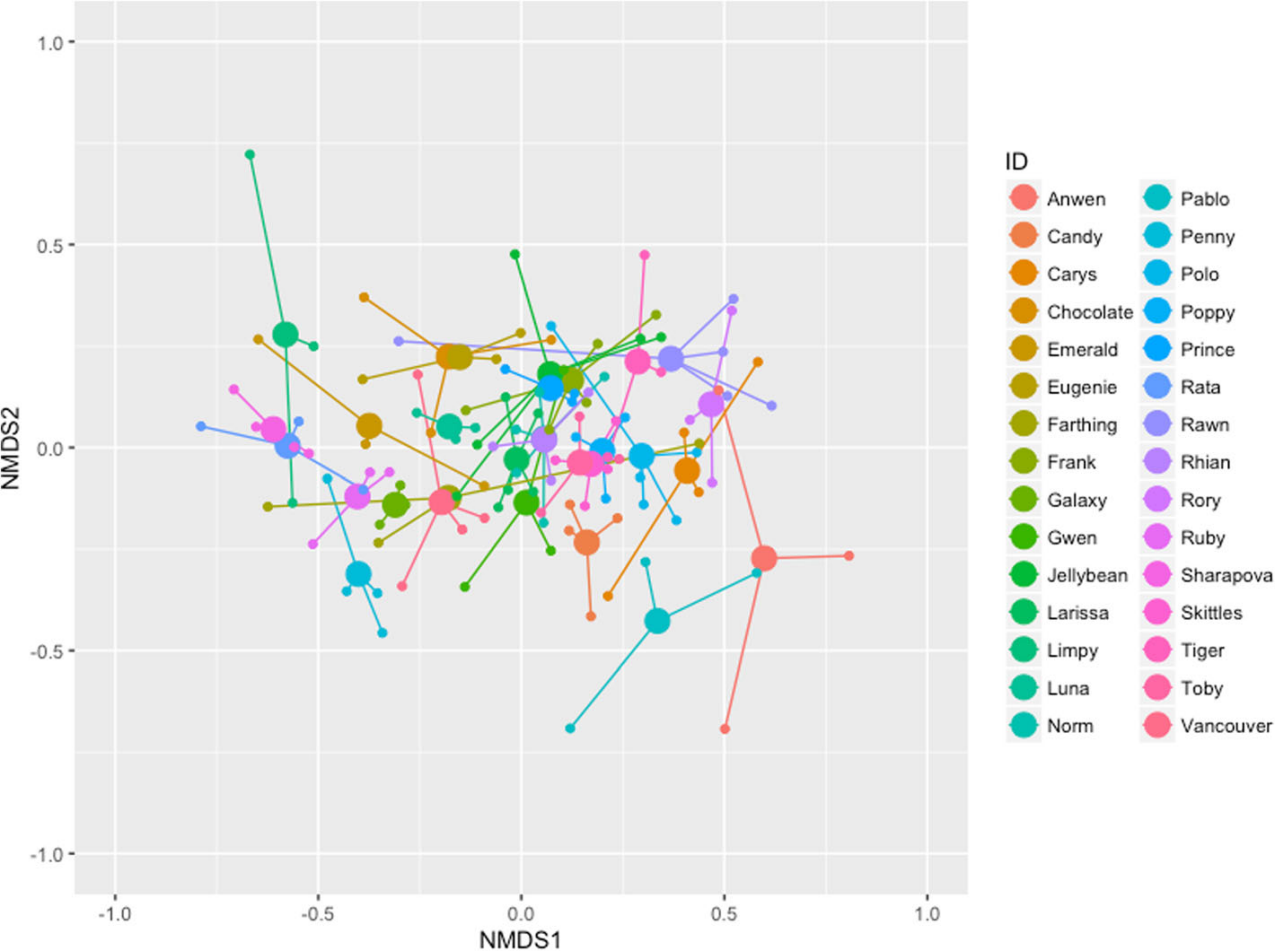
NMDS plot of the total microbiome of individual ponies in the study. Larger filled circles indicate the centroid for each individual

**Fig. 2:**
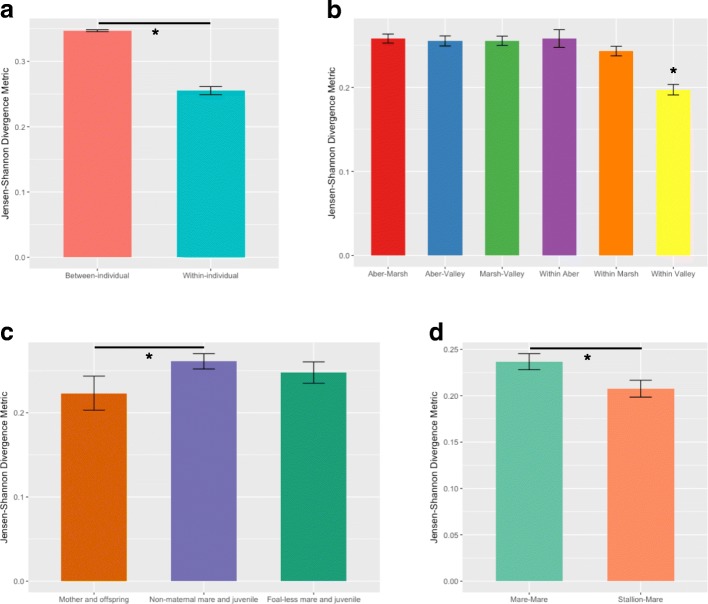
Average (± SE) Jensen-Shannon divergence metrics for pony microbiome composition **a** within and between individuals; **b** within and between bands; **c** between mothers and their offspring, as well as between juveniles and non-maternal mares and foal-less mares; and **d** between the band stallion and band mares, and between all mares within a band. Significantly different results are indicated by *

An adonis analysis showed pony ID had a significant effect on total microbiome composition (*p* < 0.001; Table 2), with 52.6% of the variation in the microbiome attributable to individual variation (Fig. 1). We obtained similar results for the adonis with the core microbiome (*p* < 0.001; Table 2), with 49.6% of the variation explained by ID. Despite the large amount of microbiome variation explained by ID, within-individual samples had significantly lower JSD values (mean of 0.255 ± 0.006) than between-individual samples (mean of 0.347 ± 0.001) (*χ*^2^ = 391.62, df = 1, *p* < 0.001) (Fig. 2a). That is, there is greater variation between individuals (average of 35%) than within individuals (average of 26%).

**Table 2:** Statistical outputs for adonis analyses of microbiome composition

Microbiome component	Adonis model	Term	*F* value	df	*R*^2^ value	% variation attributed to term	*p* value
Total	ID	ID	3.021	29. 79	0.526	52.6	0.001
Total	Band + life stage + sex	Band	2.289	2.24	0.140	14.0	0.001
Total	Band + life stage + sex	Life stage	1.708	2.24	0.104	10.4	0.011
Total	Band + life stage + sex	Sex	0.725	1.24	0.022	2.2	0.856
Core	ID	ID	3.176	29. 79	0.496	49.6	0.001
Core	Band + life stage + sex	Band	3.697	2.24	0.194	19.4	0.001
Core	Band + life stage + sex	Life stage	3.170	2.24	0.166	16.6	0.005
Core	Band + life stage + sex	Sex	0.345	1.24	0.009	0.9	0.925

**Fig. 3:**
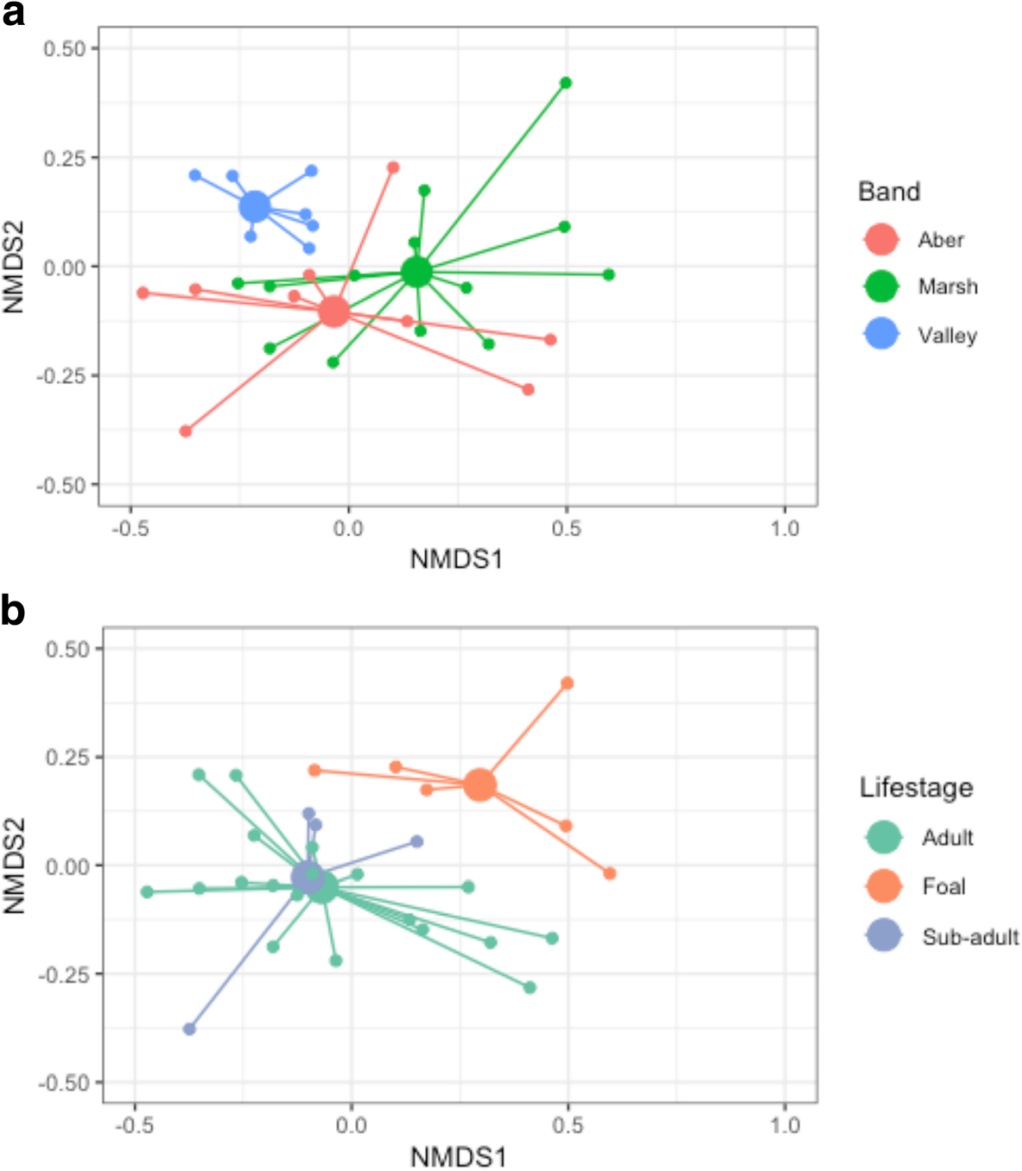
NMDS plots of the total microbiome of ponies plotted according to band membership (**a**) and life stage (**b**). Larger filled circles indicate group centroids

There were significant effects of band and life stage on total microbiome composition, but not sex (Table 2, Fig. 3a and b). In both cases, the proportion of the variation in the microbiome for these significant factors (14.0% for band and 10.4% for life stage) was much lower than for pony ID alone (52.6%). The results of the adonis analysis for the core microbiome were similar to those for the total microbiome, where band and life stage both significantly affected core microbiome composition, but sex did not (Table 2). Band and life stage account for a slightly larger proportion of the variation in the core microbiome (19.4% and 16.6%, respectively) than the total microbiome.

**Fig. 4:**
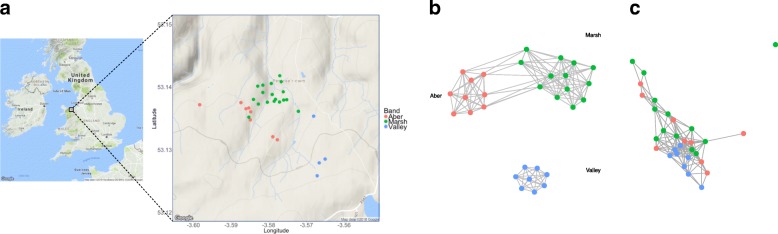
**a** Map showing the spatial distribution of pony bands encountered during sampling. **b** Social network of the sampled individuals with edge width proportional to tie strength between individuals and **c** network visualisation of microbiome distance between individuals

Consistent with the spatial distribution of the bands (Fig. 4a), microbiome composition of individuals in Valley differed considerably to those in Aber and Marsh, which are more similar to each other but still display some degree of separation (Fig. 3a). There was a significant difference in JSD metric values within and between the bands (*F*_5,424_ = 6.557, *p* < 0.001), and the Tukey posthoc indicated that within-band variation for Valley was significantly lower than the variation within the other two bands, and significantly lower than between-band variation for all three combinations (Fig. 2b). In addition to this band-level differentiation of microbiomes, there was a significant correlation between social network tie weight (i.e. spatial distribution) and microbiome composition (*τ* = − 0.11, *p* < 0.001) within bands, such that individuals that associate more have more similar microbiomes (Fig. 4b, c).

The microbiome of foals was considerably different from that of sub-adults and adults, whereas these latter two groups were very similar to one another (Fig. 3b and Additional file 1: Figure S2). An indicator analysis identified six bacterial genera (out of a possible 188) that were significantly associated (*p* < 0.05) with sub-adults compared with foals; *Prevotellaceae Ga6A1 group*, *Denitrobacterium*, *Oscillibacter*, *Anaerovibrio*, *Family_CR-115*, *and Anaerostipes*. There were no genera significantly (i.e. uniquely) associated with foals compared with sub-adults.

Maternal relationship had a significant effect on microbiome similarity (*χ*^2^ = 8.425, df = 2, *p* = 0.015; Fig. 2c). Pairwise comparisons showed significant differences in microbiome divergence between mother-offspring relationships and non-maternal mares and juveniles (foals and sub-adults combined) (*p* = 0.017). Microbiome divergences between foal-less mares and juveniles were not significantly different to those of mother-offspring relationships (*p* = 0.313) or non-maternal mares and juveniles (*p* = 1.000). Mares had significantly more similar microbiomes to the band stallion than the other mares in their band (*χ*^2^ = 4.206, df = 1, *p* = 0.040; Fig. 2d).

## Discussion

The effects of population structuring, in general, and social interactions, in particular, on microbiome composition remains poorly understood; challenges often arise in the separation of direct microbiota transmission via social interactions from effects of communal living such as a shared diet or physical environment [27, 32]. Here, we show that despite large variation between individuals in microbiome composition, spatial structuring, social relationships (i.e. mother-offspring and stallion-mare), and network ties account for microbiome similarities. The main predictor of microbiome composition is individual identity (pony ID), accounting for around 50% of microbiome variation, with ~26% variation across multiple samples collected for each individual and ~34% variation between individuals. Significant inter-individual variation in microbiome composition has been shown in other species [18, 19, 28, 55–57]. Given that such a large component of microbiome variation is due to individual ID, which individuals are present in the band (both mares and stallions) may well influence the composition of the total group microbiome.

Band membership also predicted microbiome composition, with ~ 14% of the total microbiome variation and ~ 19% of the core microbiome variation explained by this factor. That the microbiome composition of ponies belonging to Aber and Marsh are more similar to one another than Valley may be driven by both spatial structuring and diet, given that the home ranges of these bands overlap. The home ranges of Aber and Marsh are also somewhat different to that of Valley in terms of elevation, slope, and soil moisture; these are more low-lying and marshier in comparison to the steeper, more well-drained, and exposed slopes that characterised the home range of Valley during the study period. The type and quality of grasses or forage across the study area (approximately 5 km^2^) also vary according to habitat type and thus, diet quality may be driving the observed differences in bands. In addition, variation in browsing behaviour may be driving differences in microbiome composition between individuals. Dietary composition has been shown to affect the microbiome of vertebrates, with consequences for microbiome function and fitness traits such as reproductive success [37, 56, 58–60]. The microbiome of Equidae is highly susceptible to changes in diet with implications for nutrient assimilation [37] and diet can have a significant effect on population performance [61]. Microbes acquired from the environment (horizontal transfer) are likely to have greater genomic variation than vertically transmitted symbionts and thus may provide greater variation for microbiome-derived functional advantages [5]. Thus, spatial variation in microbial communities between subpopulations may have implications for fitness traits [5]. Aber and Marsh also showed higher within-band variation, comparable in magnitude to between-band variation, whereas Valley had significantly lower microbiome variation within the band. This may reflect the spatial and environmental differences experienced by members of Valley compared with Aber and Marsh, as well as fewer interactions between Valley and the other two bands. This lower microbiome variation across the group as a whole may have implications for group-level fitness. More work is required to understand how group-level microbiome variation relates to population resilience [27].

Although it may be difficult to dissociate between the influence of shared living and diet on microbiome composition *between* bands, we also identified spatial structuring of microbiome composition *within* bands, suggesting that despite communal living, social behaviours still influence microbiome composition. Social behaviours, such as grooming, that occur between members of the same band provide an opportunity for individuals to share microbial communities. Moreover, close spatial proximity also promotes the sharing of gut microbiomes through contact with recently deposited faeces, including potential coprophagy [62]. Thus, microbiomes of individuals with close social ties are more likely to converge and, indeed, our data show that specific interactions (i.e. mother-offspring and stallion-mare) lead to more similar microbiomes. This further supports the notion that individuals influence the microbiome composition of one another and ultimately the group. Affiliative behaviours occur more frequently between mothers and their offspring than between foals and non-maternal mares, but vertical transmission of microbiomes between mothers and their foals may also derive from birth and during nursing [35, 63, 64]. Ren et al. [28] also found that microbiomes of mothers and offspring were more similar to one another than between unrelated individuals in red squirrels. Interestingly, foal-less mares had an intermediate microbiome similarity to foals compared with mothers and non-maternal mares, suggesting greater levels of affiliative behaviour or social interaction between foals and mares that did not have offspring in the band. Stallions occupy a central social role in the group, unlike less well-connected mares [40], which is reflected in the greater microbiome similarity between stallions and mares (than between mares) as demonstrated here. It is not clear whether the convergence of microbiomes is driven by the stallion or the mare, but it may well be both. This may result from affiliative behaviours between stallions and mares (including mating) but may also reflect the behaviour of stallions to smell, and thus come into contact with, mares’ faeces. Given that juveniles are prone to dispersal [39, 40] and that social structures tend to break up and reform after significant events such as the annual round-up (Lea and Shultz, unpublished data), it would be interesting to follow changes in individuals’ microbiomes over such events to determine how quickly these converge and whether microbial signatures of the original band remain. It would also be of interest to compare the microbiome composition of males in bachelor groups to those of stallions to further determine the propensity for mares to alter stallion microbiomes.

Although we can estimate similarity between microbial communities across bands and individuals, we do not yet know how this relates to functional variation or fitness proxies at the subpopulation (i.e. band) level. Genetic determinants of microbiome composition, and thus the heritability of microbiomes, have been demonstrated across a range of host taxa [4, 29, 30, 57]. That band-level differences in microbiome composition were also significant in the core microbiome further supports the notion that group-level selection may occur within host microbiomes. However, dispersal of individuals between bands means that subpopulations are not genetically isolated. To further understand the potential for microbiome to act as a unit that selection can act on, it would be valuable to quantify the relative contributions of genetic, environmental, and social factors that determine microbiome composition within this system (and across a range of hosts) and to link these to fitness outcomes such as reproductive success and disease susceptibility.

We also demonstrate differences between life stages in microbiome composition of Carneddau ponies; foals had considerably different microbiome composition to both sub-adults and adults. Similar changes in microbiome composition across host development have been seen in other host organisms [10, 29, 65]. For mammals, this is particularly evident for nursing young compared with weaned individuals [66–68], and this most likely explains the results we see in our data. There was an absence of unique genera in the microbiome of foals, indicating the transition to a grass-based diet leads to the assimilation of additional bacterial groups into the gut microbiome, potentially through environmental transmission. Although gut microbiome composition has been shown to differ between sexes [69], we found that microbiomes were not significantly different between males and females for this population of semi-feral ponies. However, this may reflect a low number of males in the analysis. It would be of interest to follow changes in male microbiome across dispersion and particularly shifts in composition as stallions form new family groups and their microbiome is influenced by, and influences, new mares joining their band.

## Conclusions

Here, we show that semi-feral ponies exhibit variation in microbiome composition between bands, which may relate to social, dietary, and environmental factors. In addition, due to the high level of within-individual variation, the direction of group selection may be influenced by the individual members present in the band. Spatial structuring was also identified within bands, suggesting that despite communal living, social behaviours still influence microbiome composition. We identify two such interactions; mother-offspring and stallion-mare, that lead to more similar microbiomes, indicating that individuals influence the microbiome composition of one another and ultimately the group. Thus, we provide novel evidence that microbiome composition is structured at multiple levels within populations. The functional implications of this require further research.

## Additional files

Additional file 1: **Table S1.** Ponies from which data were collected in the study. **Figure S1.** Relative abundance of bacterial classes identified in the faecal samples of ponies belonging to the three study bands. **Figure S2.** Relative abundance of bacterial classes identified in the faecal samples of juvenile and adult ponies. **Figure S3.** Relative abundance of genera in the core microbiome. (PDF 273 kb)

Additional file 2: Microbiome analysis code as an RMarkdown file. (RMD 29 kb)

Additional file 3: Additional code for maps and network analysis. (RMD 6 kb)

Additional file 4: Sample sheet. (CSV 4 kb)
