# Supplementary material for: Gut microbiome composition is associated with spatial structuring and social interactions in semi-feral Welsh Mountain ponies
Source: Microbiome. 2018 Nov 22;6:207. doi: 10.1186/s40168-018-0593-2 (PMC6251106; doi:10.1186/s40168-018-0593-2)
Supplement: Supplementary file 1 — Table S1. Ponies from which data were collected in the study. Figure S1. Relative abundance of bacterial classes identified in the faecal samples of ponies belonging to the three study bands. Figure S2. Relative abundance of bacterial classes identified in the faecal samples of juvenile and adult ponies. Figure S3. Relative abundance of genera in the core microbiome. (PDF 273 kb) [file 40168_2018_593_MOESM1_ESM.pdf]

**Supporting Information: Spatial Structuring and Social Interactions Determine Gut Microbiome Composition in Welsh Mountain Ponies**

**Rachael E. Antwis, Jessica M. D. Lea, Bryony Unwin, Susanne Shultz**

**Table S1**

Ponies from which data were collected in the study.

| Band | Pony | Gender | Age | Year of Birth | Number of samples |
|---|---|---|---|---|---|
| Aber | Toby | Male | Stallion | 2004 | 3 |
| Aber | Carys | Female | Mare | 2007 | 4 |
| Aber | Penny | Female | Mare | 2004 | 4 |
| Aber | Rhian | Female | Mare | 2011 | 3 |
| Aber | Rory | Female | Mare | 2004 | 3 |
| Aber | Ruby | Female | Mare | 2006 | 3 |
| Aber | Vancouver | Female | Mare | 2012 | 4 |
| Aber | Pablo | Male | Subadult | 2013 | 3 |
| Aber | Eugenie | Female | Foal | 2014 | 3 |
| Marsh | Skittles | Male | Stallion | 2004 | 4 |
| Marsh | Candy | Female | Mare | 2004 | 4 |
| Marsh | Emerald | Female | Mare | 2009 | 4 |
| Marsh | Farthing | Female | Mare | 2011 | 3 |
| Marsh | Galaxy | Female | Mare | 2004 | 3 |
| Marsh | Gwen | Female | Mare | 2009 | 3 |
| Marsh | Larissa | Female | Mare | 2012 | 5 |
| Marsh | Polo | Female | Mare | 2004 | 5 |
| Marsh | Luna | Female | Subadult | 2013 | 3 |
| Marsh | Limpy | Male | Foal | 2014 | 3 |
| Marsh | Chocolate | Female | Foal | 2014 | 3 |
| Marsh | Sharapova | Female | Foal | 2014 | 4 |
| Marsh | Rata | Male | Foal | 2014 | 3 |
| Valley | Norm | Male | Stallion | 2008 | 5 |
| Valley | Anwen | Female | Mare | 2009 | 3 |
| Valley | Poppy | Female | Mare | 2011 | 3 |
| Valley | Rawn | Female | Mare | 2010 | 5 |
| Valley | Tiger | Female | Mare | 2009 | 3 |
| Valley | Jellybean | Female | Subadult | 2012 | 5 |
| Valley | Frank | Male | Subadult | 2012 | 5 |
| Valley | Prince | Male | Foal | 2014 | 3 |

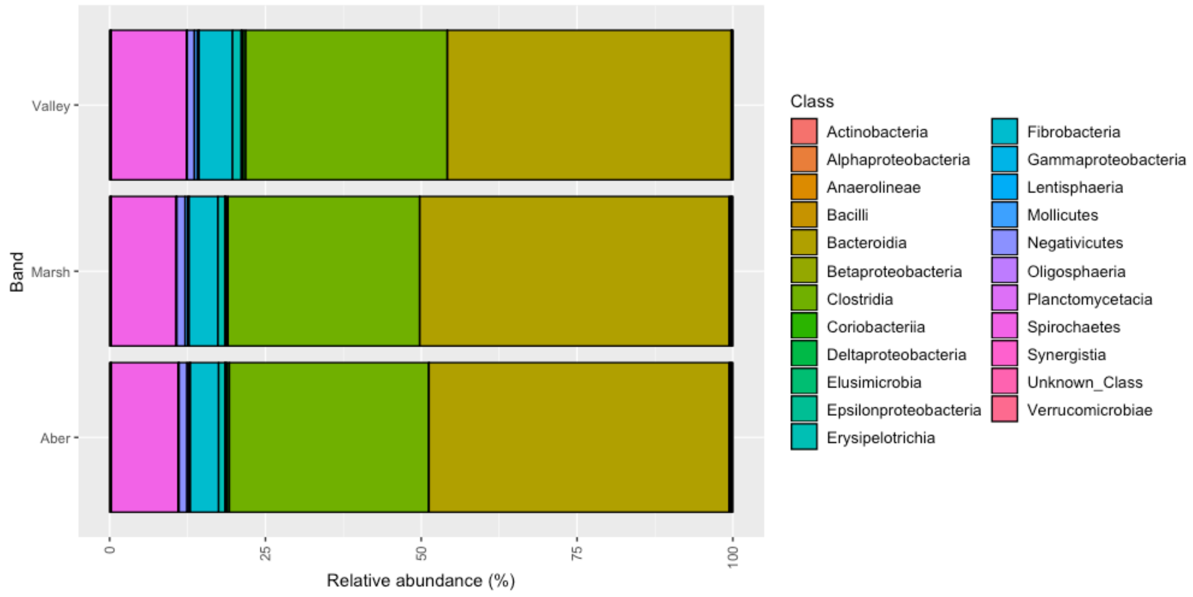


**Figure S1**

Relative abundance of bacterial classes identified in the faecal samples of ponies belonging to the three study bands.

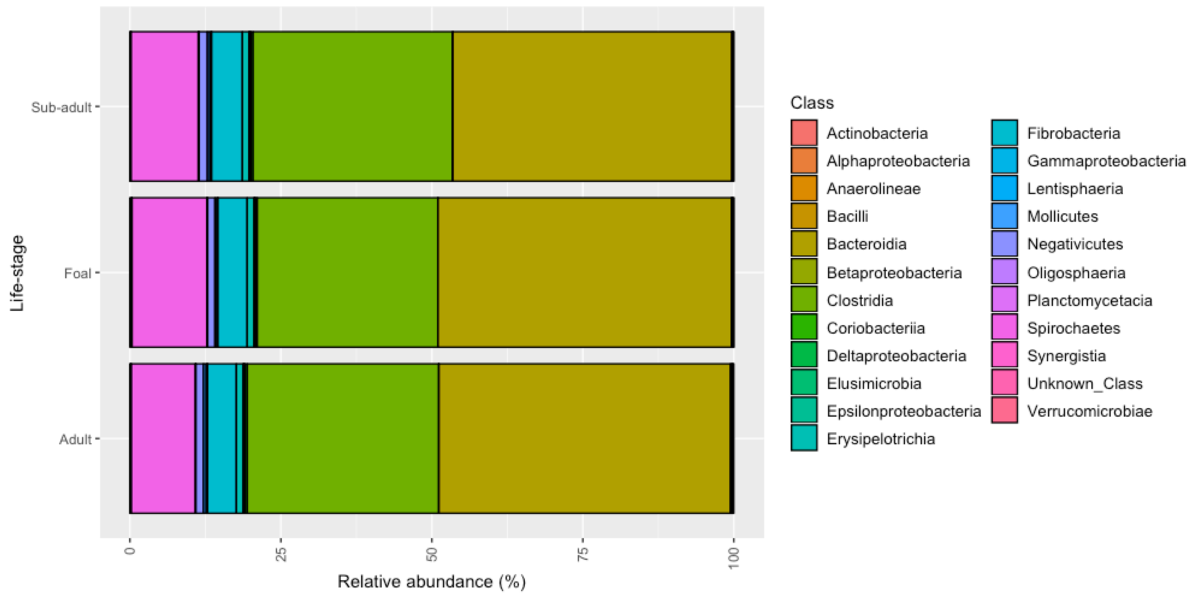


**Figure S2**

Relative abundance of bacterial classes identified in the faecal samples of juvenile and adult ponies.

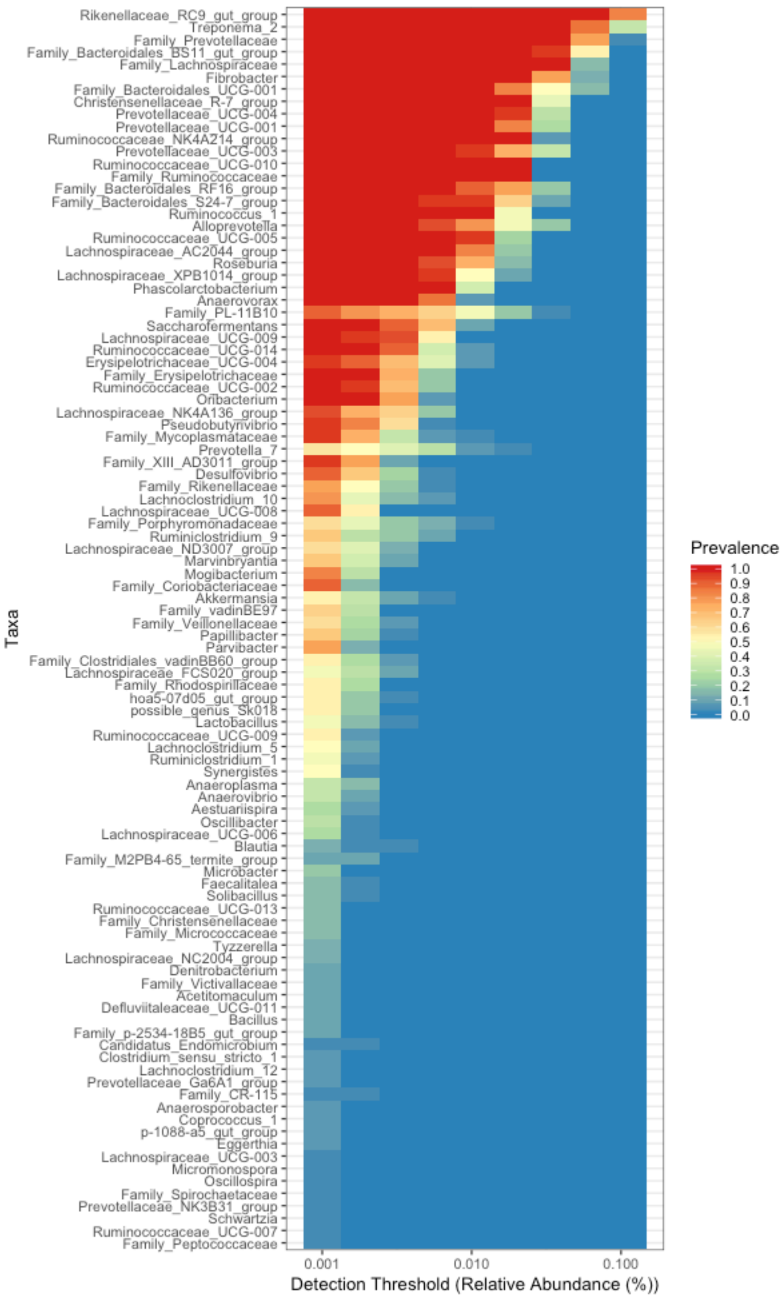


**Figure S3**

Relative abundance of genera in the core microbiome.
